# Development of High-Aspect-Ratio Soft Magnetic Microarrays for Magneto-Mechanical Actuation via Field-Induced Injection Molding

**DOI:** 10.3390/polym16213003

**Published:** 2024-10-25

**Authors:** Da Seul Shin, Jin Wook Park, Chang Woo Gal, Jina Kim, Woo Seok Yang, Seon Yeong Yang, Min Jik Kim, Ho Jae Kwak, Sang Min Park, Jong Hyun Kim

**Affiliations:** 1 Materials Processing Research Division, Korea Institute of Materials Science, 797 Changwon-daero, Seongsan-gu, Changwon 51508, Gyeongnam, Republic of Korea; jw1368park@gmail.com (J.W.P.); yus000@kims.re.kr (W.S.Y.); wook980829@kims.re.kr (S.Y.Y.); kmj4462@kims.re.kr (M.J.K.); 2 Advanced Bio and Healthcare Materials Research Division, Korea Institute of Materials Science, 797 Changwon-daero, Seongsan-gu, Changwon 51508, Gyeongnam, Republic of Korea; cwoogal@kims.re.kr; 3 Pohang Accelerator Laboratory (PLA), Pohang University of Science and Technology (POSTECH), 77, Cheongam-ro, Nam-gu, Pohang 37673, Gyeongbuk, Republic of Korea; tttjina@postech.ac.kr (J.K.); thecreated@postech.ac.kr (H.J.K.); 4 School of Mechanical Engineering, Pusan National University, 2, Busandaehak-ro 63beon-gil, Geumjeong-gu, Busan 46241, Republic of Korea; sangmin.park@pusan.ac.kr

**Keywords:** magnetoactive soft materials, soft magnetic microarrays, soft actuator, magnetorheological elastomers (MREs), field-induced injection molding

## Abstract

Magnetorheological elastomers (MREs) are in demand in the field of high-tech microindustries and nanoindustries such as biomedical applications and soft robotics due to their exquisite magneto-sensitive response. Among various MRE applications, programmable actuators are emerging as promising soft robots because of their combined advantages of excellent flexibility and precise controllability in a magnetic system. Here, we present the development of magnetically programmable soft magnetic microarray actuators through field-induced injection molding using MREs, which consist of styrene-ethylene/butylene styrene (SEBS) elastomer and carbonyl iron powder (CIP). The ratio of the CIP/SEBS matrix was designed to maximize the CIP fraction based on a critical solids loading. Further, as part of the design of the magnetization distribution in micropillar arrays, the magnetorheological response of the molten composites was analyzed using the static and dynamic viscosity results for both the on and off magnetic states, which reflected the particle dipole interaction and subsequent particle alignment during the field-induced injection molding process. To develop a high-aspect-ratio soft magnetic microarray, X-ray lithography was applied to prepare the sacrificial molds with a height-to-width ratio of 10. The alignment of the CIP was designed to achieve a parallel magnetic direction along the micropillar columns, and consequently, the micropillar arrays successfully achieved the uniform and large bending actuation of up to approximately 81° with an applied magnetic field. This study suggests that the injection molding process offers a promising manufacturing approach to build a programmable soft magnetic microarray actuator.

## 1. Introduction

Magnetoactive soft materials are a subclass of smart materials that can change their mechanical properties, shape, or functionality in response to an external magnetic field. These materials combine magnetic particles with soft, flexible matrices such as magnetorheological elastomers (MREs) or hydrogel matrices, giving them both flexibility and magneto-responsive behavior. Their unique characteristics make them ideal for innovative applications in soft robotics [1,2,3,4,5,6], biomedical devices [4,7,8,9], and soft sensors [8,10,11]. Moreover, the use of high-aspect-ratio structures enables MREs to be highly responsive to torsional and bending moments, particularly when subjected to external magnetic fields. The interaction between externally applied magnetic moments and structural torsional or bending moments in high-aspect-ratio arrays can lead to complex deformation patterns with multiple locomotions through the external magnetic stimuli. Accordingly, many researchers have developed high-aspect-ratio soft magnetic microarrays capable of magnetic-responsive actuation.

Ni, Ke, et al. [12] introduce core–shell magnetic micropillars (diameter of 6 μm and height of 80 μm) via two-step template casting technique using UV-curable polyurethane acrylate resins and Fe_3_O_4_ nanoparticles, exhibiting reprogrammable as well as programmable stimuli responsive actuation motions. They utilized elastomeric hollow shells to enable various bending motions, ranging from 5° to 45.5°, by reprogramming the spatial distribution of magnetic nanoparticles within the micropillars containing liquid resin. Park and Jeon et al. [13,14,15,16] systematically designed a variety of magnetically programmable microarrays, ranging from high-aspect-ratio microarrays to the bioinspired corolla of a flower and achiral arrays, and fabricated them to enable bending, twisting, and hybrid motions. The programmable microarrays were fabricated based on UV lithography and polydimethylsiloxane (PDMS) casting processes, while the magnetic actuation mechanics and detailed actuation angles were designed using FEM simulation. Lin, Yucai, et al. [17] applied the developed magnetic nano/micro pillar array to control microdroplet transport on the superhydrophobic surface of the arrays, achieving a maximum bending angle of 59° for moving the water droplet. Peng, Yubin, et al. [18] also presented droplet manipulation with magnetic micropillar arrays. Particularly, their hydrophobic features enabled the movement of the infusing oil via meniscus driving, demonstrating unidirectional droplet transport in a circular micropillar channel without droplet coalescence. Meanwhile, a wearable capacitive sensor was developed using a soft micromagnet array, exhibiting a high sensitivity of 1 mT and enabling touchless Morse code and Braille communication [8].

Most of the aforementioned studies have fabricated high-aspect-ratio magnetic microarrays using lab-scale fabrication methods such as PDMS casting and 3D printing, but there is a clear limitation to mass production. Given the growing attention to this field, it is necessary to develop soft magnetic microarrays used for mass production manufacturing processes, such as injection molding. Through magnetic injection molding, high-aspect-ratio magnetic microarrays can be fabricated within seconds, and the alignment of magnetic particles also can be precisely designed using an external magnetic field mold system. In our previous work, we developed stimuli-responsive flexible micropillar composites with strontium ferrite hard magnetic materials and elastomers via field-induced injection molding, confirming the potential of this process as a mass production method for fabricating high-aspect-ratio magnetic microarrays [19]. However, studies on the fabrication of injection-molded magnetic microarrays for actuator applications using soft magnetic materials have been not reported. Soft magnetic materials are indispensable in applications requiring fast response, high-frequency electromagnetic systems. Their importance is particularly high from the perspective of mass production for high-aspect-ratio magnetic microarrays, making it crucial to carry out research on mass production processes, such as injection molding, for fabricating high-aspect-ratio magnetic microarrays.

**Figure 1:**
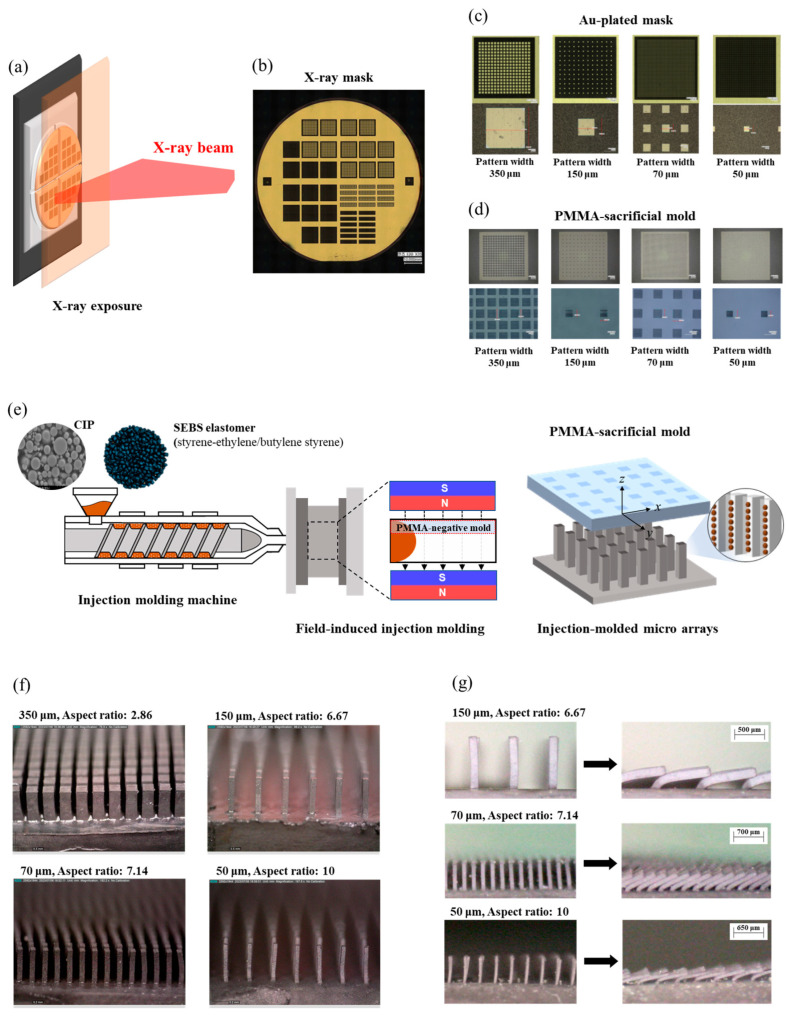
Overall manufacturing process of high-aspect-ratio soft magnetic microarrays: (**a**) Schematic illustration of X-ray beam exposure. (**b**) X-ray mask. (**c**) Details of Au-plated mask (350, 150, 70, and 50 µm pattern widths). (**d**) PMMA-sacrificial mold (350, 150, 70, and 50 µm pattern widths). (**e**) Schematic diagram of field-induced injection molding for fabricating soft magnetic microarrays with MRE, (**f**) Injection molded soft magnetic microarrays (350, 150, 70, and 50 µm pattern width with aspect ratios of 2.86, 6.67, 7.14, and 10). (**g**) Bending actuation motions of 150, 70, and 50 µm patterned microarray linear external magnetic field.

In this study, we applied an injection molding process to design a high-aspect-ratio soft magnetic microarray based on the MRE capable of magnetic-responsive actuation. The magnetic microarrays were fabricated through a two-step procedure, consisting of X-ray lithography for mold fabrication and field-induced injection molding as shown in Figure 1a–e. By utilizing X-ray lithography, we aimed to fabricate sacrificial micromolds with high aspect ratios to assess the effectiveness of the injection molding process in terms of both moldability and precision. Four types of sacrificial micromolds were fabricated with rectangular micropillars arranged in high-density arrays, featuring an interpillar spacing of 100 μm and high aspect ratios of 10 (Figure 1c,d). Subsequently, injection molding was performed with an external magnetic field applied within the mold cavity to align the magnetic particles along the direction of the pillars. The sacrificial mold was then removed through solvent debinding and successfully fabricating the microarrays with square pattern widths of 50, 70, 150, and 350 µm and corresponding heights of 500, 500, 1000, and 1000 µm, respectively, as shown in Figure 1f. The magnetic particles in the micropillars were aligned along the magnetic field direction parallel to the micropillars, exhibiting the high angular bending motion with excellent flexibility in response to the magnetic field (Figure 1g). Additionally, we analyzed the magnetorheological properties, one of the key factors in developing high-responsive microarrays, and discussed the maximum particle suspension ratio in MREs through critical solids loading analysis for the injection molding process.

## 2. Materials and Methods

**Figure 2:**
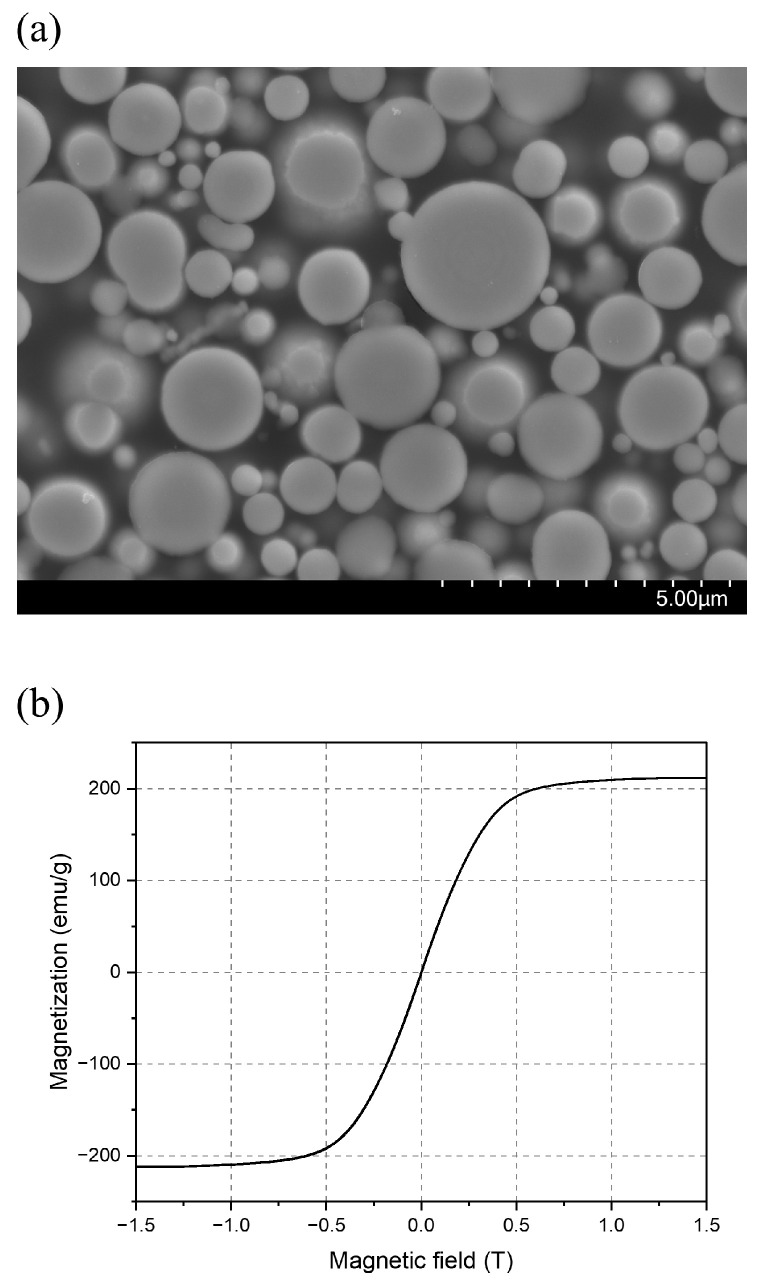
Characteristics of carbonyl iron powder (CIP, HQ grade): (**a**) particle SEM image (×5000) and (**b**) M–H hysteresis curve obtained using a vibrating sample magnetometer at 25 °C.

Carbonyl iron soft magnetic powder (CIP, HQ grade, BASF, Ludwigshafen, Germany) and a thermoplastic elastomer, styrene–ethylene/butylene–styrene (SEBS), were used as the magnetic material and polymer matrix, respectively. CIP is a representative soft magnetic powder with high permeability, low coercivity, and a narrow hysteresis loop, which indicates low energy loss during the magnetization and demagnetization cycle [20,21,22]. This ensures fast response and high efficiency in high-frequency circuits and electromagnetic applications [23]. The morphological characteristics of CIP are presented in Table 1 and Figure 2a; it has a mean particle size of 1.4 μm and a pycnometer density of 3.8 g/cm^3^. The magnetic properties and the phase structures of the powder were analyzed using a vibrating sample magnetometer (VSM, model 7407-S, LakeShore Cryotronics, Westerville, OH, USA) and an X-ray diffractometer (XRD, D-MAX/2500-PC, RIGAKU, Tokyo, Japan), respectively. Figure 2b shows the hysteresis curve of the CIP, which exhibits a narrow hysteresis loop with low coercivity (*H_c_*) and remanence (*M_r_*). For the polymer matrix, SE-130AB (styrene ethylene butylene styrene-based thermoplastic elastomer, Lotte Chemical Industry Co., Ltd., Seoul, Republic of Korea) was employed as the elastomer matrix to provide flexible and reversible motion in the micropillar arrays. Additionally, its biocompatibility and high chemical resistance made it a suitable material for applications in the biomedical device field. It showed excellent elastic recovery and high elongation at break of 850% as shown in Table 1. The detailed mechanical and physical characteristics of SE-130AB are shown in Table 1.

A torque rheometer (HAAKE PolyLab QC Lab Mixer, Thermo Scientific, Waltham, MA, USA) was utilized to measure the torque based on the solids loading of CIP and SE-130AB mixtures. The feedstock was prepared by mixing three times using a twin-screw extruder mixer at 160 °C to ensure homogeneous mixing of CIP and SE-130AB. To characterize the magnetorheological response of the feedstock, the shear viscosity and the complex viscosity were measured via the plate type rheometer (MCR 101, Anton Paar, Graz, Austria) and capillary rheometer (Rosand RH7, Malvern, UK), respectively, with the effects of temperature, strain rate, and magnetic field. The dynamic magnetorheological properties were evaluated using a frequency sweep test with the plate-type rheometer, reflecting the structural changes in the feedstock flow, including particle alignment and chain-like structure formation under an applied external magnetic field. Specifically, the magnetic response of the CIP particles in the feedstock was indirectly confirmed through dynamic shear viscosity measurements in both the on (0.1 to 0.7 T) and off (0 T) magnetic field states. These measurements included the storage modulus (*G′*), loss modulus (*G″*), and the resulting complex viscosity (η*). The frequency sweep test was conducted under an external magnetic field ranging from 0 to 0.7 Tesla, with a strain amplitude of 0.1% within the linear viscoelastic (LVE) region and an oscillation frequency range of 0.1 to 100 rad/s. The capillary rheometer was used to obtain static off-state (0 Tesla) shear viscosity at three temperatures, 160 °C, 180 °C, and 200 °C, under shear rates ranging from 100 to 10,000 s^−1^, reflecting typical injection molding conditions [24].

The fabrication of magnetic micropillar arrays was carried out by combining the following techniques: field-induced injection molding [19] and the LIGA process (German acronym: LIthographie, Galvanoformung, and Abformung) [25]. The injection molding process was conducted using a field-assisted mold system, referred to as field-induced injection molding. During the molding process, the magnetic alignment of CIP was developed through the application of an external magnetic field. The mold cavity is the square mold with dimensions of 10 mm × 10 mm in width and length and 1.5 mm in height. The used mold cavity was a square mold with dimensions of 10 mm × 10 mm (width and length) and 1.5 mm in height. The external magnetic flux was generated by two neodymium permanent magnets positioned between the cavity, allowing the flux to pass through the height (~1.5 mm) of the cavity. The magnetic flux density within the cavity was measured using a gauss-meter (FH-54, MAGNET-PHYSIK, Cologne, Germany), with an average value of 0.900 Tesla (max: 0.933 T, min: 0.791 T, standard deviation: ±0.0318).

To fabricate the micropillar arrays using field-induced injection molding, the LIGA process was applied to shape the micropatterned insert mold with a high-aspect-ratio structure (<10). The UV and X-ray lithography steps of the LIGA process were conducted at the Pohang Light Source-II (PLS-II) accelerator at Pohang University of Science and Technology, utilizing the synchrotron X-ray nanomachining/micromachining process at beamline 9D. Synchrotron X-ray was employed for precise micropatterning by masking the sacrificial polymethyl methacrylate (PMMA, Goodfellow Corp., Boulder City, NV, USA) insert mold with high-aspect-ratio structures. Thereafter, the developed PMMA mold was inserted into the 10 mm × 10 mm × 1.5 mm mold cavity within the field-assisted system, followed by the injection molding process to fill the micropatterns. The injection molding temperature was set to 160 °C. After molding, the sacrificial PMMA mold was removed through solvent debinding using acetone, followed by rinsing with deionized water. This process resulted in defect-free magnetic micropillar arrays. Details of the LIGA process and final pattern geometries are described in Section 3.2.

## 3. Results

### 3.1. Feedstock Preparation and Magnetorheological Properties

**Figure 3:**
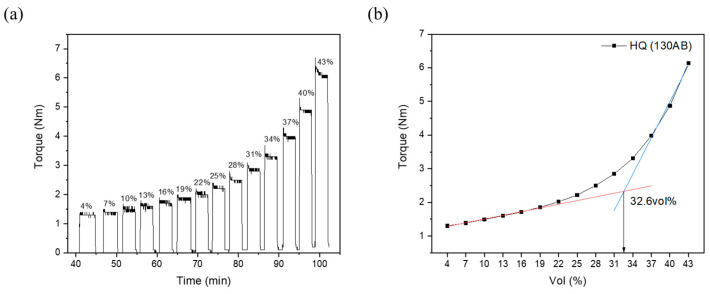
Results of torque rheometer test: (**a**) measured mixing torque for CIP powder to a SE-130AB elastomer matrix ratios ranging from 4 to 43 vol% and (**b**) average mixing torque values with corresponding critical solids loading for CIP/SE-130AB MRE.

Figure 3 shows the mixing torque of CIP/SE-130AB mixtures across a volumetric ratio of CIP loading, ranging from 4 to 43 vol% of the total volume of powder (CIP) and polymer (SE-130AB) mixtures. The mixing torque gradually increased as the CIP volume fraction increased, with the critical solids loading point occurring at the maximum rate of change in mixing torque, measured at 32.6 vol%. The critical solids loading means the closest packing composition where the powder and polymer are mixed without formation of void. Therefore, it serves as a threshold point for the injection molding process, beyond which the viscosity begins to increase infinitely due to the severe particle frictions from excess powder fraction [26]. Above the critical point, the flow index of the composition deteriorates significantly, rendering injection molding unfeasible. The critical point for the CIP/SE-130AB mixtures was measured to be approximately 32.6 vol%, and thus, we set the optimal solids loading at 30 vol%, about 3 vol% lower than the critical point, based on the recommended optimal loading condition (2 to 5 vol% lower than the critical point) for powder/binder mixture injection molding [24]. In summary, the feedstock with 30 vol% CIP addition was designed to contain the maximum powder fraction, allowing the developed micropillar arrays to achieve the maximum magnetic responsiveness during magnetic actuation.

**Figure 4:**
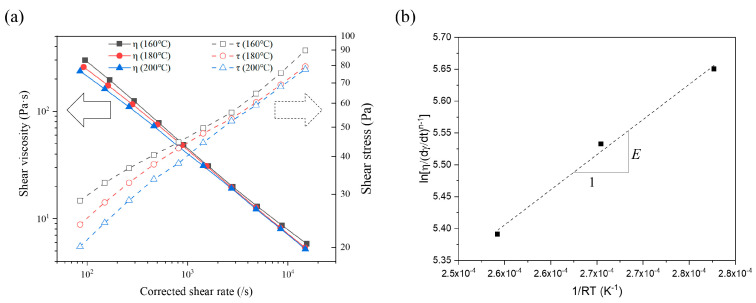
(**a**) Shear viscosity and shear stress of the 30 vol% CIP/SE-130AB feedstock at temperatures of 160, 180, and 200 °C. (**b**) Calculation of activation energy (E) for 30 vol% CIP/SE-130AB.

The rheological behaviors of the feedstock were characterized by the effects of temperature, shear rate, and magnetic field. First, the off-state (0 Tesla) shear viscosity was measured over a high shear rate range from 100 to 10,000 s^−1^ at temperatures of 160, 180, and 200 °C using a capillary rheometer, as shown in Figure 4a. The recorded shear viscosity remained below 300 Pa·s, which was within the desirable viscosity range (below 1000 Pa·s) for shear rates between 100 to 10,000 s^−1^ during molding [24]. The off-state viscosity exhibited pseudo-plastic behavior, where the molten feedstock’s shear viscosity decreased as the shear rate increased. The neat polymer, SE-130AB, also demonstrated typical pseudoplastic behavior at high shear rates due to the unraveling of polymer molecules with increasing shear rate. This pseudoplastic behavior can be described using the power law model. Additionally, the temperature sensitivity of the shear viscosity was characterized by the Arrhenius model. The combined effects of the strain rate and temperature on the rheology can be represented by these models, as given below:

(1) $$\eta \left(\gamma \right)={\dot{\gamma }}^{n-1}$$

(2) $$\eta \left(T\right)=\exp\left(\frac{E}{RT}\right)$$

where *η* is the apparent viscosity, $\dot{\gamma }$ is the shear rate, *n* is the power law exponent, *T* is the temperature, *R* is the gas constant, and *E* is the activation energy for the Arrhenius model reflecting the temperature sensitivity on the shear viscosity. A power law exponent of *n* < 1 indicates non-Newtonian fluid behavior with pseudoplasticity. The power law exponent, *n*, varied from 0.215, 0.229, and 0.257 for 160, 180, and 200 °C, respectively. Since the power law exponent, *n*, is also dependent on the temperature, it increased as the temperature increased with the linear progression relationship [27]. The Arrhenius activation energy, *E*, was determined by calculating the slope of $\ln\left(\eta /{\dot{\gamma }}^{n-1}\right)$ versus 1/RT as shown in Figure 4b. The Arrhenius activation energy, *E*, was calculated as 11,020 J/mol, which is significantly lower than that of other thermoplastic polymers, such as polypropylene (PP) and PDMS [28,29]. This result indicates that the CIP/SE-130AB feedstock is relatively insensitive to the molding temperature range of 160 to 200 °C.

**Figure 5:**
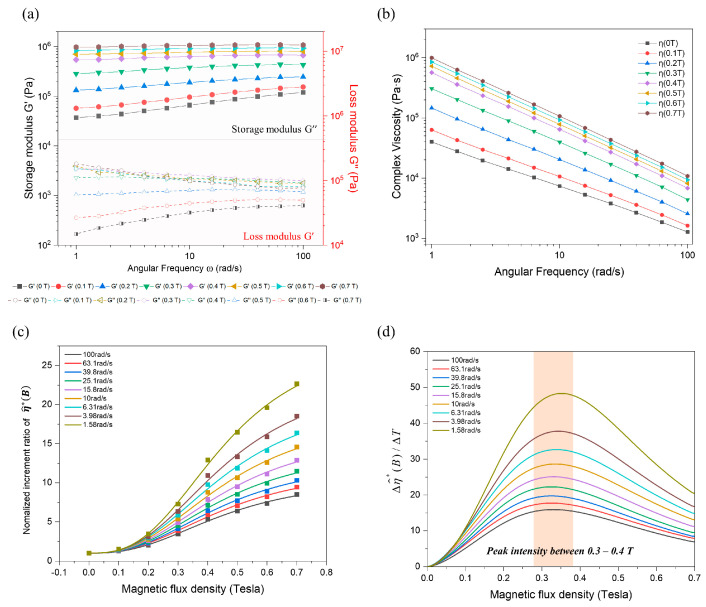
Results of frequency sweep test with external magnetic field (0 to 0.7 T) at 160 °C: (**a**) Storage modulus (*G′*) and loss modulus (*G″*) in response to angular frequency ranges from 1 to 100 rad/s. (**b**) Complex viscosity with external magnetic field from 0 to 0.7 T. (**c**) Normalized complex viscosity versus field intensity (0 to 0.7 T). (**d**) First derivative curve of normalized complex viscosity versus field intensity (0 to 0.7 T).

In Figure 5, the dynamic magnetorheological response was analyzed using a magnetic frequency sweep test that gathered the internal structure change in the polymer or feedstock system within the linear viscoelastic (LVE) region under an external magnetic field. The storage modulus (*G′*) and loss modulus (*G″*) of the feedstock at 160 °C are plotted in Figure 5a, with the external magnetic field increasing from 0 to 0.7 Tesla. The results indicate that the feedstock exhibited a higher *G′* than *G″* under both magnetic and non-magnetic field conditions, demonstrating that the CIP/SE-130AB feedstock behaved like a solid, with its elastic portion being predominant over the viscous portion. As the magnetic field increased, both the storage and loss moduli increased, as shown in Figure 5a. The strength of the dipole–dipole interactions among the magnetic powders increased with the higher magnetization, so that the magnetorheological stiffening effect increased with the increasing external field, consequently forming a chain-like structure among the particles from a macroscale viewpoint. The CIPs were magnetically aligned along the magnetic field direction and subsequently agglomerated and assembled into magnetic columnar structures due to the dipole–dipole magnetic interactions among the adjacent neighboring particles [30,31]. Within the LVE region, the CIP/SE-130AB feedstock exhibited a stable or slightly increasing *G′* as a function of angular frequency, which resulted from the extrinsically formed magnetic column structure of particle–particle interactions, as well as the intrinsically retained sturdy structure of the elastomeric matrix. Moreover, *G″* above the 0.3 T external field showed a decreasing trend, even with an increase in angular frequency, suggesting the accumulative stiffening effect of dipole interactions.

Figure 5b illustrates the results of complex viscosity (η*) with an increasing magnetic field, comparing the on-state (0.1 to 0.7 T) and off-state (0 T) dynamic shear viscosities. The complex viscosity was calculated as the complex conjugate of the elastic and viscous components [32]. η* progressively increased as the external magnetic field intensified, with the increment rate from 0 to 0.4 T being significantly higher than that from 0.4 to 0.7 T. To better understand the dynamic magnetorheological response relative to field intensity, the normalized complex viscosity (${\eta^{*}}_{off\sim 0.7T}/{\eta^{*}}_{off}$) was calculated for each angular frequency from 1.58 to 100 rad/s (Figure 5c). The normalized increment plots displayed a sigmoidal S shape, and the first derivative showed a bell-shaped increment rate (Figure 5d). The rate of increase in η* peaked in the 0.3 to 0.4 T range, then gradually decreased after this inflection point (shaded area in Figure 5d). This behavior in complex viscosity was attributed to particle alignment and the formation of columnar structures. Initially, in the magnetic field region from 0 to 0.4 T, the CIPs became magnetized and aligned along the field direction, forming chain-like structures. In this region, the CIPs rapidly magnetized due to the intrinsic low residual magnetization (0.75 emu/g). However, the chain-like structure still had a weak interaction among the particles since the external field was below the saturation magnetization field of around 0.6–0.7 T, as seen in the B–H hysteresis loop (Figure 2b). Beyond the inflection point, stronger dipole interactions were developed as the magnetization increased, leading to the formation of numerous columnar structures with more CIPs. Eventually, these interactions and the strength of the column structures reached a plateau as the field approached 0.7 T. The magnetic alignment of CIPs and the subsequent clustering mechanism in the molten feedstock were governed by three primary factors: (i) particle dipole interaction, (ii) particle–magnetic interaction, and (iii) particle–flow hydrodynamic interaction [19]. Thus, it was evident that compared with the intrinsic magnetization curve of the CIP, particle alignment and subsequent clustering formation were comparatively delayed due to the flow resistance (hydrodynamic interaction) of the molten feedstock. In other words, the degree of particle clustering in response to the magnetic field intensity was affected by the viscosity of the feedstock.

In addition, the slope of the normalized complex viscosity decreased with increasing angular frequency. At low frequencies, magnetized particles formed stable and robust chain-like structures in response to a strong external magnetic field, resulting in the maximum slope of the normalized increment ratio of complex viscosity. In contrast, as angular frequency increased, hydrodynamic interactions hindered the alignment of CIPs and the formation of column structure, leading to a decrease in the slope of the increment rate in complex viscosity [33]. In summary, the variation in complex viscosity with magnetic field was directly related to the dynamic magnetorheological response in the feedstock and could be used as an indicator for designing the magnetic alignment of micropillar arrays in MREs.

### 3.2. Development of LIGA Process and Soft Magnetic Microarrays

**Figure 6:**
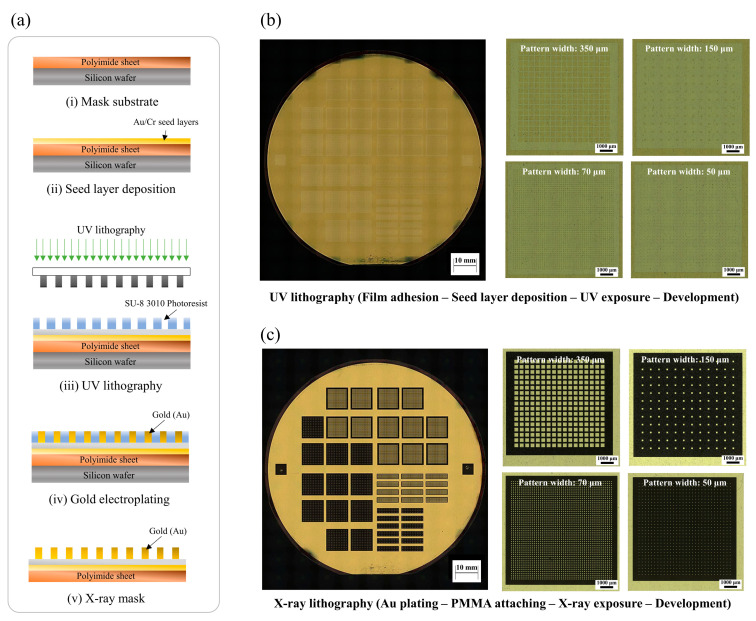
(**a**) Schematic diagram for the fabrication of the X-ray mask from (**i**) mask substrate process to (**v**) X-ray mask development. (**b**) Developed UV masks and (**c**) X-ray masks for 350, 150, 70, and 50 µm patterned PMMA molds.

The LIGA process for developing the micropatterned PMMA insert mold consisted of four steps: (1) UV photolithography, (2) X-ray mask design, (3) X-ray lithography, and (4) development. The target design of the micro pattern is listed in Table 2, with square pattern widths of 50, 70, 150, and 350 μm. Through X-ray lithography using high phonon flux energy, we achieved a high width-to-height ratio for the micropillar pattern, up to 10, with the pattern width of 50 μm. Figure 6a(i,ii) shows the intermediate photomask prior to the UV lithography process, which was made using a silicon wafer, polyimide film, Au/Cr seed layer, and SU-8 3010 (MicroChem, Newton, TX, USA). A 4-inch silicon wafer was prepared as the base substrate for the photomask [25]. A 450 μm photosensitive polyimide film was adhered on top of the silicon wafer, acting as a transmission layer with high transparency. Next, the seed layer, consisting of Au and Cr, was deposited on the polyimide film. Prior to UV lithography, a negative-type UV photoresist, SU-8 3010, was coated onto the seed layer. The SU-8 3010 solution was spin-coated at speeds ranging from 300 to 1000 rpm, resulting in an approximately 10 to 16 μm thick negative photoresist layer.

**Figure 7:**
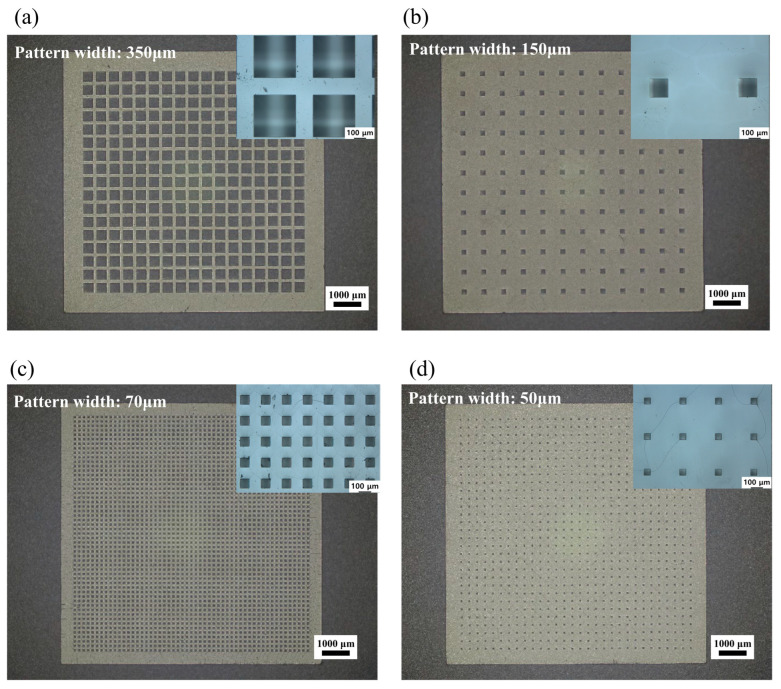
OM images of the top view of PMMA insert molds: (**a**) Pattern width, 350 μm; aspect ratio, 2.86. (**b**) Pattern width, 150 μm; aspect ratio, 6.67. (**c**) Pattern width, 70 μm; aspect ratio, 7.14. (**d**) Pattern width, 50 μm; aspect ratio, 10.

**Figure 8:**
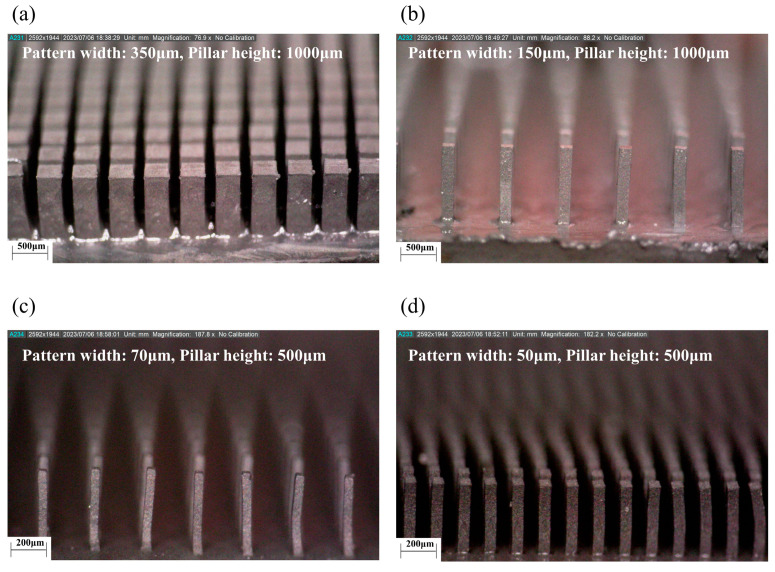
The 3-D OM images of 30 vol% CIP/130-AB soft magnetic microarrays: (**a**) Pattern width, 350 μm; aspect ratio, 2.86. (**b**) Pattern width, 150 μm; aspect ratio, 6.67. (**c**) Pattern width, 70 μm; aspect ratio, 7.14. (**d**) Pattern width, 50 μm; aspect ratio, 10.

In the first step, the SU-8 3010 layer was exposed to a UV light source through the working mask, as shown in Figure 6a(iii). Since SU-8 3010 was a negative photoresist, the exposed structure underwent cross-linking and strengthening during lithography. The SU-8 3010 layer was then baked at 65 °C for 1 min, followed by a successive heating at 95 °C for 5 min. The non-exposed portions were dissolved by SU-8 liquid developers, and intermediate UV photomasks are shown in Figure 6b. In the second step, a gold layer was electroplated onto the SU-8 3010 layer of the photomask to serve as an X-ray absorber as shown in Figure 6a(iv). Au plating was performed on the SU-8 3010 pattern to develop the X-ray absorber. Au is widely used as an X-ray absorber material due to its extremely low transmittance for X-ray irradiation [34]. The gold layer was plated to a thickness of around 10 to 15 μm. Figure 6c shows the final X-ray gold mask for the X-ray LIGA process. Next, the positive photoresist PMMA was prepared for the sacrificial insert mold. PMMA sheets with thicknesses of 500 and 1000 μm were attached to a graphite substrate. Subsequently, X-ray lithography was performed through the X-ray gold mask onto the positive photoresist PMMA mold. The target X-ray exposure energy was set to 4.5 kJ/cm^3^. After X-ray exposure, the exposed PMMA was etched using a GG developer in the fourth step. The GG developer consisted of 5 vol% 2-aminoethanol, 15 vol% deionized water, 20 vol% tetrahydro-1, 4-oxazine, and 60 vol% 2-(2-butoxyethoxy) ethanol. The sacrificial PMMA insert molds, shown in Figure 7, exhibited no visible defects. The micropatterns of the PMMA molds had pattern widths ranging from 50 to 350 μm, with sheet thicknesses of 500 and 1000 μm, achieving aspect ratios of up to 10. Table 2 provides detailed information on the micropattern insert molds, including the square pattern width, height, and interpillar spacing. Using the developed sacrificial PMMA molds in Figure 7, the mold insert injection molding process was carried out under an external magnetic field aligned along the micropillar direction. The field intensity within the mold cavity was set to approximately 0.9 T, a strength sufficient to align the particles along the micropillars, surpassing the peak gradient of 0.3 to 0.4 T, as well as the plateau field intensity of 0.7 T as shown in Figure 5c,d. After the field-induced injection molding into the insert mold, the PMMA insert molds were removed in the solvent debinding step. The molded samples were immersed in 99.5% acetone solution for 3 h at room temperature, allowing them to chemically dissolve and depolymerize. Figure 8 presents 3-D optical microscope images of the high-aspect-ratio soft magnetic microarrays.

**Figure 9:**
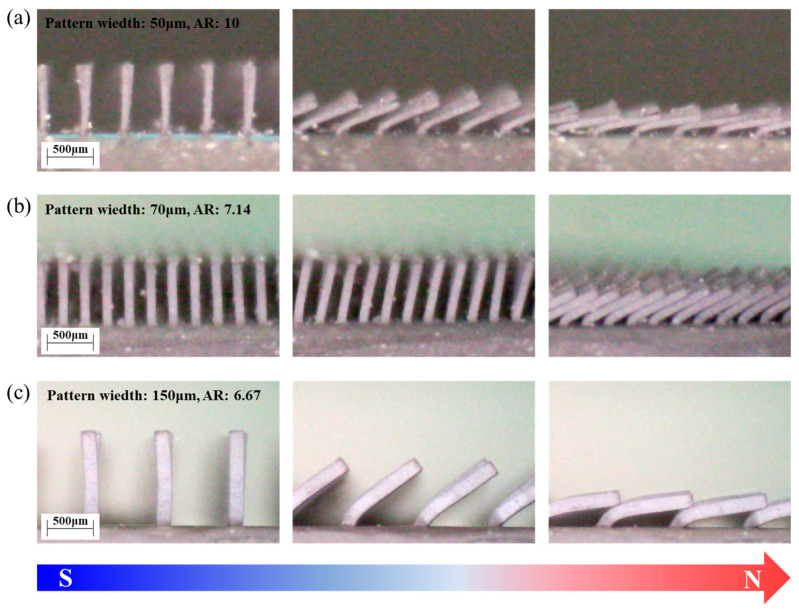
Bending motion of micropillar arrays under an external field of 0.5 T: (**a**) pattern width, 50 μm; aspect ratio, 10; (**b**) pattern width, 70 μm; aspect ratio, 7.14; (**c**) pattern width, 150 μm; aspect ratio, 6.67.

An external magnetic field of 0.5 to 0.6 T was applied to induce the magneto-mechanical responses (referred to as magnetic actuation) of the developed magnetic microarrays. As shown in Figure 9, the microarrays exhibited bending motion in response to the linear external magnetic field. The particle chain structures along the axial direction of the arrays generated magnetic dipole moments in the presence of the magnetic gradient field, producing magnetic bending torques along the linear direction of the external field. When a gradient magnetic field was applied to the magnetic microarrays, the resulting magnetic body force per unit volume generated by the soft-magnetic particles could be expressed as

(3) F=∇B⋅M

where ∇*B* is the gradient of the external magnetic field, and *M* is the magnetic dipole moments of the magnetic suspensions. Soft magnetic materials, such as CIP, are highly responsive to external magnetic fields due to their low coercivity (*H_c_*), which is the force required to demagnetize the material. Additionally, their narrow hysteresis loop allows them to be easily magnetized and demagnetized in alignment with the applied magnetic field [35,36]. In addition to single bending actuation, we confirmed that by switching the external magnetic field on and off, the soft magnetic microarrays exhibited reversible actuation motion, demonstrating the possibility of reversible shape reconfiguration (see Supplementary Video S1). Our developed soft magnetic microarrays with square pattern widths of 50, 70, and 150 μm and aspect ratios of 10.00, 7.14, and 6.67 exhibited bending angles of 79, 65, and 81°, respectively. This was attributed to the highly responsive magneto-mechanical bending moments inherent in the high-aspect-ratio microarray structures. In addition, further research from a manufacturing perspective was necessary to develop a viable mass production process for fabricating injection-molded soft magnetic microarrays. This would include the use of permanent molds in place of sacrificial molds to enhance production efficiency. Therefore, this aspect will be addressed in future studies focusing on the mass production approach via the injection molding process.

## 4. Discussion

The high-aspect-ratio soft magnetic microarrays were manufactured using an optimized ratio of MRE consisting of carbonyl iron powder (CIP) and styrene-ethylene/butylene-styrene (SEBS) elastomer via field-induced injection molding. Through critical solids loading analysis, we determined the critical solids loading of the CIP/SEBS-based MRE to be 32.3 vol%, and a maximum injectable volume fraction of 30 vol% CIP was adopted for CIP/SE-130AB feedstock fabrication. Additionally, the static and dynamic magnetorheological behaviors, such as static shear viscosity and complex viscosity, were characterized using capillary and plate-type rheometers. The magnetorheological interaction was analyzed to determine the relationship between the CIP alignment and the intensity of the induced magnetic field. The magnetorheological response, based on the first derivative of complex viscosity (η*), peaked at around 0.3–0.4 T, indicating the highest rate of dipole moment and particle cluster chain formation. This suggested that a critical magnetic field intensity was required to align the particles during molten flow in the MRE during injection molding. To fabricate the high-aspect-ratio structures, a PMMA sacrificial mold was produced using X-ray lithography, and the mold was inserted into a cavity for field-induced injection molding with an external field of 0.9 T. The soft magnetic microarrays, with pattern widths of 350, 150, 70, and 50 μm, heights of 1000, 1000, 500, and 1000 μm, and aspect ratios of 2.86, 6.67, 7.14, and 10, were fabricated without any visible defects. The developed soft magnetic microarrays exhibited bending motion with a maximum angle of 81° in response to the external magnetic field and demonstrated reversible shape reconfiguration at on/off external stimuli. In order to assess the durability of the microarrays and the elastic recovery of the structures during continuous motions, a comprehensive evaluation of the mechanical properties of the MREs, such as tensile and bending tests, is essential. These detailed mechanical assessments should be explored in future research. In summary, this research indicates that the injection molding process offers a viable manufacturing method for constructing high-aspect-ratio magnetic actuators.

## Figures and Tables

**Table 1:** Characteristics of the carbonyl iron powder and styrene-ethylene/butylene styrene (SEBS, SE-130AB) elastomer.

**Carbonyl Iron Powder (CIP, HQ Grade)**	**Physical Properties**		**Magnetic Properties**			
	**Average Particle Diameter (APD)**	**Pycnometer** **Density**	**Residual Induction (*M_r_*)**	**Intrinsic Coercive Force (*H_Ci_*)**	**Positive** **Magnetization (*M_S, positive_*)**	**Negative** **Magnetization (*M_S, negative_*)**
	1.4 μm	3.8 g/cm^3^	0.75 emu/g	11.60 Oe	−212.12 emu/g	212.99 emu/g
**Styrene-ethylene/Butylene Styrene (SEBS-130AB) Elastomer**	**Physical Properties**		**Mechanical Properties**			
	**Hardness**	**Density**	**Tensile Strength (Break)**	**Elongation (Break)**	**100% Modulus**	**Tear Stress**
	30 shore A	0.9 g/cm^3^	4.4 MPa	850%	1.2 MPa	13 kN/m

**Table 2:** Dimensional information of PMMA micropatterned mold.

Square Patterns Size (µm)	Pillar Spacing (µm)	Pillar Height (µm)	Aspect Ratio (Height to Pattern Width)
350	150	1000	2.86
150	600	1000	6.67
70	100	500	7.14
50	250	500	10.00

## Data Availability

The original contributions presented in the study are included in the article, further inquiries can be directed to the corresponding author.

## Supplementary Materials

The following supporting information can be downloaded at https://www.mdpi.com/article/10.3390/polym16213003/s1: Video S1: Bending actuation motions at on/off the external stimuli.
